# Le paludisme congénital maladie à *Plasmodium falciparum*: aspects épidémiologiques, cliniques, biologiques, thérapeutiques et pronostiques à Ouagadougou, Burkina Faso

**DOI:** 10.11604/pamj.2014.18.47.3614

**Published:** 2014-05-13

**Authors:** Kisito Nagalo, Fousséni Dao, Philippe Minodier, Oumarou Sawadogo, Harouna Sanon, François Housséini Tall, Diarra Yé

**Affiliations:** 1 Service de Pédiatrie Clinique El Fateh-Suka, Ouagadougou, Burkina Faso; 2 UFR/Sciences de la Santé, Université de Ouagadougou, Burkina Faso; 3 Service de Pédiatrie, Urgences Enfants CHU Nord, Marseille, France; 4 Programme National de Lutte contre le Paludisme, Ouagadougou, Burkina Faso

**Keywords:** Paludisme, Plasmodium falciparum, congénital, nouveau-né

## Abstract

**Introduction:**

Le paludisme reste un problème majeur de santé publique en Afrique subsaharienne. Le but de l’étude était d’étudier le paludisme congénital maladie et les objectifs étaient de déterminer la prévalence du paludisme congénital maladie, de décrire sa présentation clinique et biologique, et de calculer son taux de mortalité.

**Méthodes:**

Une étude rétrospective cas-témoins sur une période de 10 années (de Juin 1999 à Mai 2009) était menée dans l'Unité de Néonatologie de la Clinique El Fateh-Suka. Etaient inclus tous les nouveau-nés âgés de moins de sept jours hospitalisés qui avaient une recherche documentée du *Plasmodium* par la goutte épaisse et le frottis sanguin.

**Résultats:**

La fréquence du paludisme congénital maladie était 170/697 (24,4%). Parmi les facteurs maternels, un nombre de grossesses ≥ 2 était associé au paludisme congénital maladie (OR = 1,93; IC95% [1,03-3,63]; p = 0,04). Aucun facteur propre au nouveau-né n’était associé à la maladie. La fréquence de la fièvre était 48,3%, les autres signes cliniques évoquaient une infection néonatale. *Plasmodium falciparum* était la seule espèce parasitaire en cause. Les décès survenaient dans 55% des cas dans les 24 heures suivant l'admission et le taux de létalité était de 20/170 (11,8%).

**Conclusion:**

La prévalence du paludisme congénital maladie est élevée dans notre Unité et les facteurs de risque de la maladie sont difficiles à cerner. Parce que les symptômes ne sont pas spécifiques, la recherche de Plasmodium doit être systématique chez tout nouveau-né malade en zone d'endémie palustre.

## Introduction

Selon les estimations de l'Organisation Mondiale de la Santé (OMS), il y avait en 2010, 219 millions de cas de paludisme qui étaient à l'origine de 660 000 décès dans le Monde; l'Afrique concentrait 91% de la mortalité et 86% des décès concernaient des enfants de moins de cinq ans [1]. Le paludisme congénital est secondaire à la transmission transplacentaire du *Plasmodium*. Il est défini par la présence de formes asexuées du parasite dans le sang périphérique du nouveau-né dans les sept premiers jours de vie [2]. On en distingue deux formes: i) le paludisme congénital infestation (PCI), défini par la présence du *Plasmodium* dans le sang du cordon ou sur sang périphérique chez un nouveau-né asymptomatique âgé de moins de sept jours et ii) le paludisme congénital maladie (PCM) où le nouveau-né est symptomatique [3, 4]. Sa prévalence qui varie selon les régions est imparfaitement connue en zone d'endémie palustre [4]. Le Burkina Faso est un pays de la zone sahélienne de l'Afrique peuplé de 16 millions d'habitants. En 2010, le paludisme y était la première cause de mortalité des enfants de moins de 5 ans (24%), devant la pneumonie (18%), la diarrhée (12%) et la prématurité (9%) [5]. Dans la capitale Ouagadougou, le paludisme sévit sur un mode d'endémie stable avec une recrudescence saisonnière durant la période de mai à octobre. Jusqu'ici, aucune étude à notre connaissance n'avait été menée sur le PCM au Burkina Faso. Le but de cette étude était de décrire les aspects épidémiologiques, cliniques, biologiques, thérapeutiques et pronostiques du PCM.

## Méthodes

### Cadre d’étude

L’étude s'est déroulée à la Clinique El Fateh-Suka (CFS), un établissement de santé privé situé à Ouagadougou qui possède un service de pédiatrie avec une Unité de Néonatologie (UN). Celle-ci reçoit les nouveau-nés nés à la maternité de l’établissement et ceux référés des autres centres de santé de la ville, parfois d'autres régions du Burkina Faso.

### Type, période et population d’étude

Il s'agissait d'une étude rétrospective menée à partir des dossiers de tous les nouveau-nés hospitalisés entre le 15 juin 1999 et le 14 juin 2009, soit une période de 10 ans. Tous les nouveau-nés de moins de sept jours de vie qui avaient une recherche confirmée du *Plasmodium* par la goutte épaisse et le frottis sanguin et qui étaient hospitalisés pendant la période d’étude étaient inclus. Les nouveau-nés âgés de plus de sept jours, ceux qui n'avaient pas bénéficié d'une goutte épaisse et d'un frottis sanguin et ceux dont le résultat de la goutte épaisse et du frottis sanguin n’était pas documenté étaient exclus de l’étude. Les nouveau-nés dont la goutte épaisse et le frottis sanguin étaient négatifs (les témoins) servaient de contrôles pour la comparaison des fréquences avec les nouveau-nés dont la goutte épaisse et le frottis sanguin étaient positifs (les cas).

### Recueil et analyse des données

Une fiche de collecte conçue pour l’étude a permis de recueillir, quand elles étaient disponibles, les informations sur i) la mère (âge, nombre de grossesses, nombre d'accouchements, nombre de consultations prénatales (CPN) et les pathologies survenues au cours de la grossesse), ii) l'accouchement (rupture des membranes, voie d'accouchement), iii) le nouveau-né (sexe, terme, poids, taille, périmètres crânien et thoracique, score d'Apgar, symptômes cliniques), iv) les examens complémentaires disponibles (goutte épaisse/frottis sanguin, hémogramme, hémoculture, coproculture, uroculture, CRP..), v) le traitement, vi) le mode de sortie (vivant, décès, contre avis médical), vii) le(s) diagnostic(s) de sortie. Les données étaient ensuite saisies à l'aide du logiciel Epi-Info 7.0 puis analysées grâce à SPSS 16.0. Pour les aspects descriptifs de l'analyse, les distributions des fréquences étaient générées pour toutes les variables. Seuls les cas où les variables étaient valides étaient analysés, ce qui explique la fluctuation de l’échantillon en fonction de la variable étudiée. Pour les variables quantitatives, la moyenne était calculée avec l’écart-type. Le test du chi-deux ou le test exact de Fischer (pour les effectifs inférieurs à 5) permettait de comparer les proportions des variables qualitatives. Pour rechercher une association entre le PCM et un facteur de risque, l'Odds ratio (OR) avec un intervalle de confiance à 95% (IC95%) était calculé. Les tests statistiques donnant des valeurs de *p* < 0,05 étaient considérés comme statistiquement significatifs.

### Diagnostic de paludisme congénital maladie

Le prélèvement pour la goutte épaisse et le frottis sanguin était fait au talon du nouveau-né. Les tests étaient pratiqués selon les techniques classiques et lus par un microbiologiste du laboratoire de la CFS. Le diagnostic de PCM était posé devant la découverte de formes asexuées de *Plasmodium* chez un nouveau-né âgé de moins de sept jours hospitalisé.

### Autres définitions

Selon leur âge, les mères étaient rangées en deux groupes: “≤ 27 ans” et “> 27 ans”. Un nombre de CPN < 04 était dit faible, normal s'il était ≥ 04. Le prématuré était un nouveau-né d’‘âge gestationnel < 37 semaines d'aménorrhée (SA) révolues et l'hypotrophe (ou faible poids de naissance) désignait un poids de naissance < 2500 g. La fièvre était définie pour une température corporelle > 37,5 °C, l'anémie pour un taux d'hémoglobine < 150 g/L de sang et l'hypoglycémie pour une glycémie < 02 mmol/L. Le niveau de la parasitémie était classé en “faible” (densité parasitaire < 1000 trophozoïtes de *Plasmodium falciparum*/µL) et “élevé” (densité parasitaire ≥ 1000 trophozoïtes de *Plasmodium falciparum*/µL).

## Résultats

### Résultats généraux

En dix ans, 697 nouveau-nés ont été hospitalisés dans l'UN de la CFS. Parmi eux, 324 répondaient aux critères d'inclusion et 373 étaient exclus. Chez les 324 nouveau-nés inclus, la goutte épaisse était positive chez 170 et négative chez les 154 autres. La prévalence du paludisme congénital maladie était donc de 170/697 (24,4%) au sein des nouveau-nés de l'UN de la CFS.

### Données maternelles et périnatales

Les nouveau-nés étaient nés de mères âgées en moyenne de 27,9 ± 5,8 ans (minima 17, maxima 41 ans) avec une médiane de 27 ans. Le nombre moyen de grossesses était de 2,5 ± 1,3 par femme (minima 1, maxima 6 grossesses) et celui des accouchements de 2,1 ± 1,3 par femme (maxima 6 accouchements). Le nombre moyen de CPN effectuées par femme était 4,0 ± 1,2 (minima 2, maxima 7 CPN). L’âge gestationnel moyen à la naissance des nouveau-nés était 37,9 ± 3,2 SA (minima 28, maxima 43 SA). Dans le Tableau 1 qui présente les caractéristiques maternelles et périnatales chez les nouveau-nés selon le diagnostic de PCM, le seul facteur significativement associé au PCM était le nombre de grossesses. En effet, les nouveau-nés dont les mères avaient plus d'une grossesse couraient 1,93 plus le risque de développer un PCM que ceux dont les mères étaient primigestes (OR = 1,93; IC95% (1,03-3,63); p = 0,04). Les autres facteurs, à savoir l’âge et la parité de la mère, le nombre de CPN, la rupture des membranes avant l'accouchement, la présence d'une pathologie pendant la grossesse, la voie d'accouchement, l’âge gestationnel du nouveau-né n’étaient pas associés au PCM. Par ailleurs, il n'y avait pas de lien statistique entre le nombre de grossesses et la parasitémie du nouveau-né, le niveau de la densité parasitaire des nouveau-nés de mères primigestes était comparable à celui de leurs homologues nés de mères multigestes.

**Tableau 1:** Caractéristiques maternelles et périnatales chez les nouveau-nés selon le diagnostic du paludisme congénial maladie (PCM) à Ouagadougou, 1999-2009, Burkina Faso

Caractéristiques maternelles et périnatales	Nouveau-nés avec PCM (%)	Nouveau-nés sans PCM (%)	OR [IC_95%_]	*p*
**Age de la mère (ans) (n = 137)**				
≤ 27	30 (46,9)	43 (58,9)	1,62 [0,82-3,20]	0,16
> 27	34 (53,1)	30 (41,1)		
**Nombre de grossesses (n = 181)**				
≥ 2	66 (74,2)	55 (59,8)	1,93 [1,03-3,63]	0,04
< 2	23 (25,8)	37 (40,2)		
Nombre de CPN*(n = 105)				
< 4	18 (36,7)	21 (37,5)	1,03 [0,47-2,28]	0,93
≥ 4	31 (63,3)	35 (62,5)		
**Pathologie sur grossesse (n = 145)**				
Oui	60 (88,2)	59 (76,6)	2,29 [0,92-5,67]	0,07
Non	8 (11,8)	18 (23,4)		
**Age gestationnel (SA) (n = 264)**				
< 37	40 (29,9)	30 (23,1)	1,42 [0,82-2,46]	0,21
≥ 37	94 (70,1)	100 (76,9)		
**Voie d'accouchement (n = 291)**				
Voie basse	91 (61,1)	89 (62,7)	0,93 [0,58-1,50]	0,78
Césarienne	58 (38,9)	53 (37,3)		
**RPM** † **(n = 98)**				
Oui	29 (53,7)	18 (40,9)	1,67 [0,75-3,75]	0,21
Non	25 (46,3)	26 (59,1)		

* Consultations Prénatales

† Rupture Prématurée des membranes

### Données démographiques, cliniques et biologiques

Le sex ratio était de 1,3, l’âge moyen à l'admission des nouveau-nés 0,98 jour et le poids moyen de naissance 2674 ± 700 g (minima 1130, maxima 4500 g). La température moyenne à l'admission était 37,5 ± 1,3 °C (minima 34, maxima 40,8°C) et la fièvre était plus fréquente de manière statistiquement significative chez les nouveau-nés sans PCM (62,2%) que chez ceux qui avaient le PCM (48,3%) (OR = 1,77; IC95% (1,09-2,85); p = 0,02). L'hémogramme montrait un taux moyen d'hémoglobine de 146 ± 2,7 g/L (minima 72, maxima 209 g/L) et une glycémie moyenne de 3,3 ± 2,2 mmol/L (minima 0,5, maxima 17,1 mmol/L). Le poids de naissance, la coloration, la respiration, le tonus, les réflexes archaïques, l'anémie et l'hypoglycémie n’étaient pas statistiquement associés au PCM (Tableau 2). *Plasmodium falciparum* était la seule espèce plasmodiale identifiée, avec une densité moyenne parasitaire de 1091 ± 2825 trophozoïtes/µL (minima 16, maxima 30000 trophozoïtes/µL).

**Tableau 2:** Signes cliniques et biologiques observés chez les nouveau-nés selon le diagnostic de paludisme congénital maladie (PCM) à Ouagadougou, 1999-2009, Burkina Faso

Signes cliniques et biologiques	Nouveau-nés avec PCM (%)	Nouveau-nés sans PCM (%)	OR [IC 95_%_]	*p*
**Poids de naissance (g) (n = 293)**				
< 2 500	58 (38,4)	41 (29,1)	1,50 [0,92-2,45]	0,10
≥ 2 500	94 (61,6)	100 (70,9)		
**Température (°C) (n = 278)**				
Fièvre	69 (48,3)	84 (62,2)	1,77 [1,09-2,85]	0,02
Pas de fièvre	74 (51,7)	51 (37,8)		
**Coloration (n = 320)**				
Anormale*	51 (30,5)	53 (34,6)	0,83 [0,52-1,32]	0,44
Normale	116 (69,5)	100 (65,4)		
**Respiration (n = 308)**				
Détresse respiratoire	29 (18,1)	30 (20,3)	0,87 [0,49-1,54]	0,63
Pas de détresse respiratoire	131 (81,9)	118 (79,7)		
**Tonus (n = 322)**				
Anormal†	54 (32,0)	43 (28,1)	1,20 [0,74-1,94]	0,45
Normal	115 (68,0)	110 (71,9)		
**Réflexes archaïques (n = 322)**				
Anormaux‡	57 (33,7)	43 (28,1)	1,30 [0,81-2,10]	0,28
Normaux	112 (66,3)	110 (71,9)		
**Hémoglobine (g/L) (n = 281)**				
< 15,0	88 (61,1)	74 (54,0)	1,34 [0,83-2,15]	0,23
≥ 15,0	56 (38,9)	63 (46,0)		
**Glycémie (mmol/L) (n = 154)**				
< 2,0	16 (21,0)	10 (12,8)	1,81 [0,76-4,30]	0,17
≥ 2,0	60 (79,0)	68 (87,2)		

* Cyanose, pâleur, teint grisâtre

† Hypotonie, hypertonie

‡ Faibles, absents

### Infections associées au paludisme congénital maladie

Les infections bactériennes confirmées étaient constituées de 02 bactériémies (l'une à *Proteus penneri*, l'autre à *Staphylococus aureus*). Les entérites aiguës, au nombre de 11, étaient dues à *Escherichia coli* (08 cas), *Pseudomonas aeruginosa* (02 cas) et *Klebsiella oxytoca* (01 cas). Il y avait 01 méningite purulente aseptique et 04 infections néonatales bactériennes présumées.

### Traitement et évolution

Le traitement n’était pas documenté chez 11 cas de PCM soit 6,5%. Chez les 159 autres, le traitement antipaludique administré par la voie parentérale était à base de quinine en intraveineuse (135 cas; 84,9%) ou d'artémether en intramusculaire (10 cas; 6,3%). Le traitement par la voie orale était fait par l'amodiaquine (06 cas; 3,8%), les combinaisons thérapeutiques à base d'artémisinine (05 cas; 15,1%) et la chloroquine (03 cas; 1,9%). Une transfusion sanguine était réalisée dans 07 cas (4,4%). Une antibiothérapie était associée au traitement antipaludique dans les cas d'infections bactériennes intercurrentes. La durée moyenne d'hospitalisation était 5,9 jours (minima 01, maxima 67 jours), la maxima était observée chez un nouveau-né prématurissime. Vingt (11,8%) des cas de PCM sont décédés, en moyenne 4,8 jours après leur admission. Il n'y avait pas de décès parmi les cas de co-infections PCM-infection bactérienne. Les décès survenaient dans 55% des cas dans les premières 24 heures suivant l'admission.

## Discussion

### De la méthodologie et des limites de l’étude

Notre étude semble être celle qui rapporte le plus grand nombre d'enfants avec PCM dans la littérature à notre connaissance. Son caractère rétrospectif n'a cependant pas permis un recueil exhaustif des données car certaines manquaient dans les dossiers cliniques des patients. De plus, il s'agissait d'une étude hospitalière, menée de surcroît dans une structure privée, ce qui a pu introduire un biais de sélection des patients. Nonobstant ces réserves, cette étude nous a permis d'analyser les données épidémiologiques, cliniques, biologiques, thérapeutiques et pronostiques du PCM dans notre UN.

### De l’épidémiologie du paludisme congénital maladie

Le paludisme congénital était considéré jusqu’à récemment comme une entité nosologique rare, même en zone d'endémie palustre. En effet, la transmission materno-fœtale d'anticorps anti-palustres [6], la présence de l'hémoglobine fœtale chez le bébé [7], le jeune âge et le milieu enzymatique des hématies du nouveau-né qui sont défavorables au développement du parasite [8] sont des facteurs qui joueraient un rôle protecteur vis-à-vis du paludisme. Si des *Plasmodium* peuvent être transmis par la mère à son enfant pendant la grossesse, 70% d'entre eux seraient détruits deux à trois jours après la naissance de celui-ci [9, 10]. De ce fait, le PCI est la forme de paludisme congénital la plus fréquemment rapportée dans de nombreuses études [11]. Ils ne resteraient alors que 7 à 10% [9], voire seulement 1,7% [3] les nouveau-nés parasités qui développent la maladie et des études plus anciennes mentionnaient déjà la relative rareté des cas symptomatiques du paludisme congénital [12–14].

En contraste de notre étude, des prévalences plus faibles de PCM sont rapportées en Afrique de l'Ouest où on note 1,7% au Togo [3] alors qu'au Nigéria, ce sont 2 à 6,9% qui sont rapportées [9, 15–18]. La variabilité de prévalence observée d'une étude à l'autre résiderait dans: 1) des différences dans la définition même du paludisme congénital; 2) le niveau d'immunité maternelle; 3) le type d’échantillon de sang examiné (sang périphérique des nouveau-nés ou sang de cordon); 4) l'expérience des techniciens dans les examens du frottis sanguin et de la goutte épaisse; 5) la méthode de détection du parasite (coloration de Giemsa ou polymerase chain reaction (PCR); ou même 6) de véritables différences environnementales [19]. Dans notre étude, il y avait beaucoup plus de cas de PCM parmi les nouveau-nés des femmes multigestes que parmi ceux des primigestes. L'on s'attendait pourtant à observer le contraire car les primigestes sont beaucoup plus susceptibles que les multi-gestes de contracter le paludisme, de développer un paludisme placentaire et de transmettre la maladie à leur fœtus. En effet, des études montrent que le paludisme est plus fréquent chez la primigeste que chez la multigeste à cause d'une immunité acquise par la femme au fil des grossesses qui protégerait son bébé [8, 20–24]. Bien que n'ayant pas pu vérifier la présence ou non du paludisme chez les mères dans notre étude, notre constat semble cependant être supporté par les résultats d'une étude kényane. Cette étude a, sur une cohorte de 453 enfants, montré que le paludisme placentaire réduisait le risque de parasitémie au cours de la première année de vie chez le nourrisson de mère primigeste et les auteurs concluaient que le paludisme placentaire diminuait le risque de parasitémie parmi la progéniture des primigestes mais augmentait le risque chez celle des multi-gestes [25]. La multigestité serait donc un facteur qui accroît le risque de paludisme aussi bien chez le nouveau-né que chez le nourrisson. Nous n'avons pu évaluer la qualité de la prévention anti paludique chez les femmes du fait du caractère rétrospectif de notre étude. Ainsi, nous ne savons pas si les femmes prenaient effectivement le traitement préventif intermittent par la sulfadoxine-pyriméthamine adopté par le Burkina Faso à partir de 2005 ou la chimioprophylaxie par la chloroquine dans les années antérieures. L'utilisation d'une moustiquaire imprégnée d'insecticide à longue durée d'action et les mesures collectives de lutte anti vectorielle étaient-elles effectivement observées par les femmes à domicile? Sans doute que rassurées par plusieurs grossesses (surtout si elles se sont bien déroulées), les multi-gestes ne prenaient-elles plus assez de précautions par rapport aux primigestes qui n'en étaient qu’à leur toute première expérience? Autant de questions auxquelles notre étude n'a pu répondre. Nous n'avons pas non plus exploré le statut immunitaire des femmes alors que l'on sait que l’état immunitaire de la femme enceinte est également évoqué comme un facteur susceptible d'influencer la transmission materno-fœtale du paludisme et donc son expression chez le nouveau-né. Par exemple, la co-infection par le virus de l'immunodéficience humaine augmenterait la densité parasitaire placentaire et la transmission du paludisme transplacentaire [26]. Des études prenant en compte tous les facteurs ci-dessus évoqués seront utiles pour une meilleure compréhension du paludisme congénital.

### Des aspects cliniques et biologiques du paludisme congénital maladie

Dans le paludisme congénital maladie, la fièvre a une fréquence variable qui va de rare (8,4%) [10] à constante (100%) [16, 18], les différences entre les études pouvant être dues au mode de recrutement des patients. Avec moins de la moitié des nouveau-nés qui étaient fébriles dans notre étude, le PCM paraît simuler une infection néonatale bactérienne. En effet, plutôt que par la fièvre, les infections néonatales bactériennes se manifestent beaucoup plus par des troubles neurologiques et hémodynamiques. Dans notre série, les troubles neurologiques (troubles du tonus et des réflexes archaïques) étaient plus fréquents chez les nouveau-nés avec PCM que chez leurs homologues sans PCM, même si la différence n’était pas significative. En général, ils étaient cependant moins fréquents qu'au Togo où sur les 40 cas de PCM rapportés, ils étaient omniprésents, constitués par l'hyporéactivité (18 cas), les convulsions (13 cas), l'hypotonie (12 cas), le coma (07 cas) [3], la différence étant encore sans doute due à la petite taille de l’échantillon de cette étude. De façon moins marquée, d'autres troubles neurologiques tels que le refus de téter (10,8%), les pleurs incessants (8,1%) sont décrits dans le PCM [24] mais c'est bien plus tard dans sa deuxième semaine de vie que l'irritabilité et la faible succion seraient prédictives de paludisme congénital chez le nouveau-né [27]. La fréquence des troubles de la coloration n’était pas statistiquement différente entre les deux groupes de nouveau-nés, même si elle était légèrement plus importante chez ceux sans PCM que chez les impaludés où près du tiers (30,5%) de ces derniers avait une coloration cutanée anormale. Ces troubles hémodynamiques sont plus fréquents au Togo où ils sont rapportés dans 90% des cas de PCM, avec la cyanose qui est notée dans 16 des 40 cas [3]. Les nouveau-nés avec PCM étaient plus fréquemment anémiés que leurs homologues sans PCM mais la différence n’était pas statistiquement significative. Cette fréquence de l'anémie parmi nos cas était plus importante que celle dans cette étude togolaise où ne sont rapportés que 22,5% de cas d'anémie. La fréquence de l'anémie est aussi faible au Nigeria avec 15,7% de cas mais cette étude s'est basée sur le taux d'hématocrite pour définir l'anémie à la naissance [18]. En zone d'endémie palustre, l'anémie du nouveau-né est étroitement liée à celle de sa mère, les deux principaux facteurs de survenue de l'anémie chez celle-ci étant la malnutrition (en particulier la déficience en fer) et le paludisme gestationnel [28]. En général, comme dans notre série, l'anémie est peu sévère, nécessitant rarement une transfusion sanguine [3, 29].

Dans notre étude, *Plasmodium falciparum* était la seule espèce parasitaire en cause et cette observation est conforme aux données nationales [30, 31] et sous régionales ouest-africaines [3, 16, 17] qui attestent que c'est l'espèce plasmodiale la plus répandue. La parasitémie chez les patients dans notre étude était en moyenne plus élevée que celle rapportée dans d'autres études en Afrique de l'Ouest. En effet, au Nigéria, de très faibles densités parasitaires de 128 [17], 8 à 200 [18], 17 à 2940 [23] et 47 à 1019 parasites/µL [32] sont rapportées tandis qu'au Togo la parasitémie varie de 700 à 3000 parasites/µL [3]. Cette différence est sans doute liée à des différences environnementales ou aux protocoles locaux de traitement antipaludique appliqués.

### Des aspects thérapeutiques et pronostiques du paludisme congénital maladie

Nos patients étaient traités plus souvent par la quinine en intraveineuse en raison du potentiel de gravité du paludisme chez le nouveau-né [33, 34]. Les dérivés de l'artémisinine étaient peu utilisés car d'apparition plus récente et la chloroquine est aujourd'hui abandonnée. La mortalité due au paludisme congénital varie selon les études. Elle est faible voire nulle au Nigéria [9, 16, 35] et peut atteindre 25% dans certaines études où les nouveau-nés cumulaient en fait des facteurs de risque tels que prématurité, hypotrophie et anémie [3]. L'importante mortalité observée dans notre étude (11,8%) n’était pas influencée par les infections associées au PCM et elle était par ailleurs le reflet d'une activité hospitalière où la qualité de la prise en charge des nouveau-nés pouvait être améliorée. Bien entendu, ce taux de mortalité ne reflète pas la situation de terrain où beaucoup de femmes et d'enfants n'ont pas accès aux soins de base dans un pays pauvre comme le Burkina Faso. Nous n'avons pas pu évaluer le taux de séquelles à long terme des nouveau-nés, mais il est fort plausible qu'un paludisme des premiers jours de vie peut causer une incapacité permanente, par trouble neurologique par exemple.

## Conclusion

Notre étude montre que le paludisme congénital maladie est fréquent dans notre Unité. En zone d'endémie palustre, chez un nouveau-né malade, la goutte épaisse et le frottis sanguin doivent être systématiques afin de ne pas méconnaître un paludisme congénital maladie car ses symptômes ne sont pas spécifiques. Notre étude montre aussi que les facteurs de risque de la maladie sont difficiles à cerner. Le contrôle du paludisme congénital passe par une meilleure prévention de celui de la femme enceinte grâce au renforcement des moyens existants, en attendant la mise à disposition d'un vaccin efficace.
